# A Case of Success: Guidelines-Based Treatment to Control Atrial Fibrillation-Induced Cardiomyopathy—Atrioventricular Node Ablation and Cardiac Resynchronization Therapy to the Rescue

**DOI:** 10.3390/reports8030150

**Published:** 2025-08-20

**Authors:** Neda Jonaitienė, Grytė Ramantauskaitė, Jolanta Laukaitienė

**Affiliations:** 1 Department of Cardiology, Medical Academy, Lithuanian University of Health Sciences, Eivenių St. 2, LT-50161 Kaunas, Lithuania; gryte.ramantauskaite@kaunoklinikos.lt (G.R.); jolanta.laukaitiene@kaunoklinikos.lt (J.L.); 2 Lithuanian Society of Cardiology, Eivenių St. 2, LT-50161 Kaunas, Lithuania

**Keywords:** heart failure, atrial fibrillation, optimal treatment, reduced ejection fraction

## Abstract

**Background and Clinical Significance**: Heart failure with reduced ejection fraction (HFrEF) and atrial fibrillation (AF) frequently coexist, creating a complex clinical interplay that exacerbates morbidity and mortality. AF can directly precipitate or worsen HFrEF through mechanisms such as tachycardia-induced cardiomyopathy, loss of atrial contribution to ventricular filling, and irregular ventricular response. The use of evidence-based therapies improves clinical outcomes in patients with HFrEF. **Case Presentation**: We present a clinical case of a 58-year-old man with left bundle branch block (LBBB), tachysystolic AF, and the aforementioned induced HFrEF. The patient’s medical treatment was optimized according to recent guidelines. Subsequent to the improvements in HF treatment, the patient’s echocardiographic data showed a higher left ventricle ejection fraction (LVEF); however, it remained below 35%. Moreover, tachysystolia persisted and was not sufficiently controlled with medications. Therefore, an upgrade of the pacemaker to cardiac resynchronization therapy (CRT) following the destruction of the AV node was performed to control tachysystolic AF and worsening of HF. After the treatment adjustments, the patient’s symptoms regressed, and echocardiography showed improved LVEF up to 41%. **Conclusions**: This case highlights the successful identification and timely application of intensive heart rate control management and heart failure induced by AF treatment.

## 1. Introduction and Clinical Significance

Heart failure (HF) induced by atrial fibrillation (AF) remains one of the greatest challenges in modern cardiology [1]. Despite the latest treatment options, the management and treatment of these patients remains challenging. In complicated cases like these, clinical guidelines can help to assign appropriate treatment options and ensure a high quality of care—this is a crucial step as patients do not always receive the appropriate treatment, especially in challenging cases like this. Conventional, guideline-based treatment and management strategies should be considered to achieve the best possible outcomes, including conventional HF treatment and appropriate AF management through rhythm and rate control, as outlined in the latest CARE-AF framework [2]. We present a case of successful guideline adaptation in real clinical practice—a case of a patient with AF-induced HF, tachycardia, and distinctly impaired quality of life. Following appropriate, step-by-step, guideline-based treatment, the patient’s symptoms regressed and his quality of life improved significantly—this could be a good example of successful application of guideline-directed therapy in other similar cases.

## 2. Case Presentation

**Figure 1:**
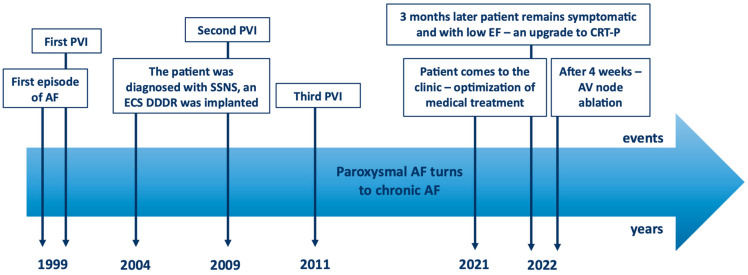
The timeline of the events. AF—atrial fibrillation; AV—atrioventricular; CRT-P—cardiac resynchronization therapy - pacemaker; ECS—electrocardiostimulator; EF—ejection fraction; PVI—pulmonary vein isolation; SSNS—sick sinus node syndrome.

A 58-year-old man attended a cardiologist consultation because of palpitations, dyspnea, and tachycardia. Consequently, his symptoms were classified as New York Heart Association functional Class III HF. In 2004, the patient was diagnosed with sick sinus node syndrome (SSNS), and a DDDR-type heart pacemaker was implanted. The patient has had permanent AF since the age of 35. He has had multiple pulmonary vein isolation (PVI) procedures, one in 1999, another in 2009, and a third in 2011, but these procedures ended in failure with the recurrence of AF after each procedure. After a few years, his AF was considered permanent, as there were no more episodes of recurrence of the sinus rhythm. Throughout his medical history, HF symptoms, such as dyspnea and intolerance to physical exertion, were observed. He was prescribed amiodarone earlier, but that was discontinued due to limited effect on rhythm control. The timeline of the case is summarized in Figure 1.

**Figure 2:**
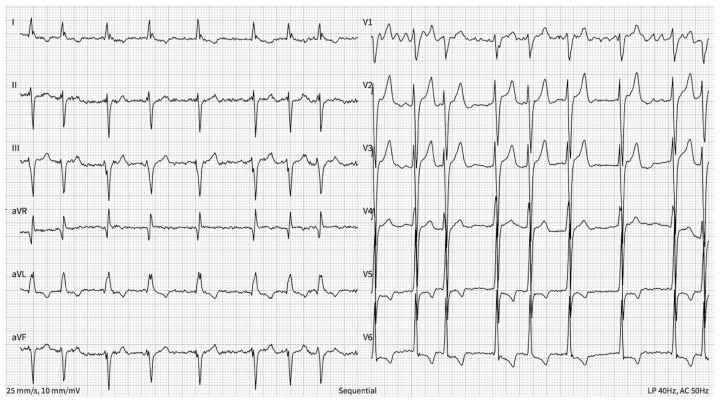
Electrocardiogram at the time of outpatient admission. The electrocardiogram shows atrial fibrillation with QRS morphology that complies with left bundle branch block, with a QRS duration of 122 ms, an increased heart rate of about 97 bpm, and T-wave inversion in I, aVL, V5 and V6.

Since then, the patient has had permanent AF with frequent episodes of tachycardia, with his heart rhythm rising to about 140–160 bpm during mild physical exertion. The electrocardiogram (ECG) revealed AF, left bundle branch block (LBBB) with a QRS duration of 122 ms, and an increased heart rate of about 97 bpm and T-wave inversion in V5 and V6 (Figure 2). The echocardiography showed a significantly reduced left ventricle ejection fraction (LVEF) of 15% and eccentric LV hypertrophy with the left atrium volume measuring 90 milliliters and the left ventricle end-diastolic volume (LVEDV) measuring 160 milliliters. There were signs of mild aortic, mitral, and tricuspid regurgitation. The patient was using only beta-blockers (metoprolol succinate 47.5 mg twice a day) for heart rate control along with Rivaroxaban 20 mg once a day for thromboembolic event prevention and no other HF medications. During the consultation, the patient was prescribed an increased dose of metoprolol. Due to persistent tachycardia, the dosage was increased to 75 mg twice a day. In addition, due to the significantly reduced ejection fraction, sacubitril/valsartan 24/26 mg twice a day was introduced, and the dose was uptitrated to 49/51 mg twice a day over four weeks (a greater increase was not possible due to hypotension). Dapagliflozin 10 mg once a day and Spironolactone 25 mg once a day (the dose was uptitrated to 50 mg a day over four weeks as there were no contraindications—the patient’s glomerular filtration rate was 80.9 mL/min/1.73 m^2^ and his serum potassium concentration was 4.01 mmol/L) were also introduced. Coronary angiography was performed—there were no stenoses in the coronary arteries.

After three months of optimized HF treatment, the patient came for a follow-up visit—his LVEF had improved (his LVEF was 24%) but remained lower than 35% and he remained in chronic AF with frequent episodes of tachycardia upon minimal exertion. Uptitration of beta-blockers was not an option because of hypotension. Due to low LVEF and tachycardia, an upgrade of a pacemaker to cardiac resynchronization therapy (CRT) was planned, and after that, the destruction of the atrioventricular (AV) node to control tachycardia. The procedures were successfully performed with a 4-week break between them.

**Figure 3:**
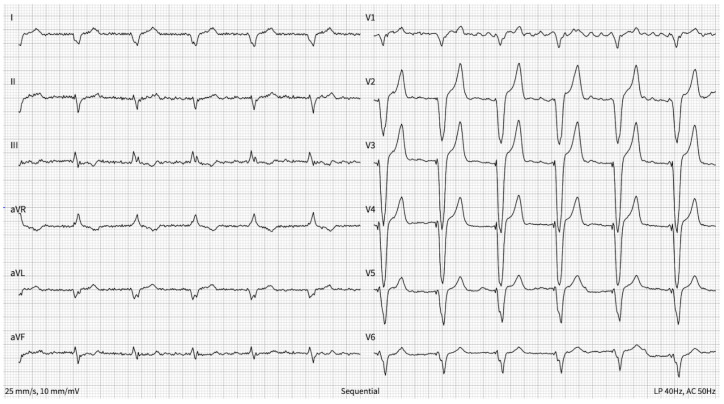
Electrocardiogram after CRT implantation and atrioventricular node ablation. The electrocardiogram shows atrial fibrillation, effective ventricular stimulation with a QRS duration of 162 ms, and a heart rate of 60 bpm.

After the procedures, the patient came for a control visit after three months. The patient had no complaints, and he had no signs of dyspnea or palpitations. His quality of life had improved—he could work and complete his usual everyday and free-time activities without any remaining symptoms—and his HF was evaluated as NYHA I functional class. The ECG showed AF and stimulated QRS complexes, and his heart rate was 60 bpm (Figure 3). In the ECG, QRS complexes were enlarged—this is because in the first ECG, they were the patient’s own heartbeats, and now, all of the beats were stimulated due to them being wider than normal. The echocardiography showed improved LVEF—it was 41% (improved from 15%). The regurgitation of the tricuspid valve remained mild, and there were no signs of right heart dysfunction. Signs of eccentric hypertrophy remained, but the LVEDV was lower—121 milliliters. Laboratory tests were also performed—the brain natriuretic peptide (BNP) concentration was 48.3 ng/L (normal value < 26.5 ng/L), and that of high-sensitivity Troponin I (hs-TnI) was 27.4 ng/L (normal value < 31 ng/L). The function of the CRT was checked—the pacemaker was functioning, and the expected battery lifetime was about 10 years. Prescribed medications were continued, and a control visit to a cardiologist and control of CRT parameters and battery lifetime once a year was recommended. To date (for 2 years after implantation), the patient remains in a stable condition, his HF remains in the NYHA 1 functional class, and his heart function has not deteriorated and remains stable.

## 3. Discussion

**Figure 4:**
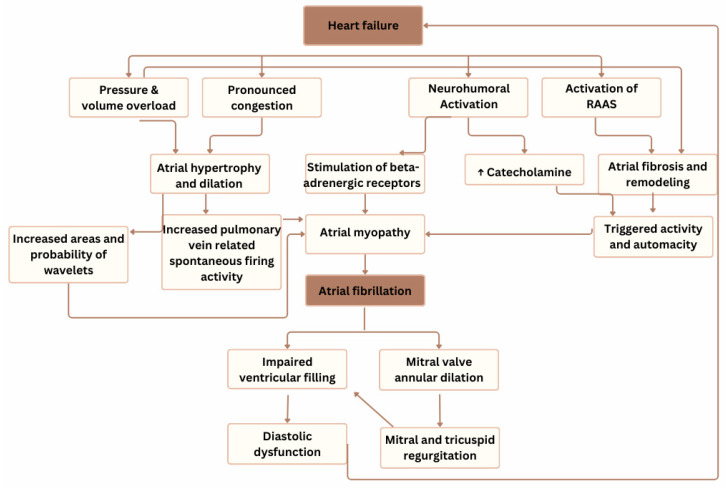
The pathophysiological interaction between heart failure and atrial fibrillation. RAAS—renin–angiotensin–aldosterone system, ↑—an increase.

HF is an escalating health problem associated with substantial morbidity and mortality rates. AF is frequently associated with HF and may contribute to its onset or progression through several pathophysiological mechanisms, including tachycardia-induced cardiomyopathy, loss of atrial contribution to ventricular filling, and irregular ventricular response [3]. The pathophysiological mechanisms between HF and AF are shown in Figure 4.

There are several mechanisms through which HF can promote AF development: in HF with reduced EF, chronic pressure and volume overload often lead to atrial hypertrophy and subsequent dilation (eccentric left atrial remodeling). This atrial dilation and more pronounced congestion are associated with increased pulmonary vein-related spontaneous ectopic activity [4]. This provides an increased area in which the re-entrant wavelets can exist, increasing the likelihood of AF development [5]. Activation of the neurohumoral system can elevate catecholamine levels, leading to stimulation of beta-adrenergic receptors, which may contribute to the onset of AF [6]. Similarly, activation of the renin–angiotensin–aldosterone system (RAAS) promotes changes within the atria that encourage the development of AF [7]. Therefore, the suppression of the RAAS system has been shown to reduce the development of AF in patients with HF [8].

However, AF can contribute to the development and exacerbation of HF through several mechanisms: increased heart rate and irregular rhythm impair ventricular filling, and the loss of effective atrial contraction compromises diastolic function [9]. It is also known that arrhythmia can cause so-called tachycardia-induced cardiomyopathy, which is a form of non-familial dilated cardiomyopathy. The pathophysiology of arrhythmia-induced cardiomyopathy includes lowering systolic contractility and cardiac output due to persistent tachycardia, increasing myocardial wall tension and causing left ventricular dilation. The diagnosis of tachycardia-induced cardiomyopathy is confirmed if left ventricular systolic volume (LVSD) is completely (or partially if there is any pre-existing heart disease) reversible within only a few weeks or months of successful treatment of arrhythmia [10]. In our patient’s case, the LVEF recovered from 15% to 41%. The recovery was partial, supposedly due to long-lasting AF with tachycardia episodes, which can cause myocardial remodeling and fibrosis in the LV. This results in wall thinning and ventricle dilation and lowers the chance of full LVEF recovery [11,12]. However, the possibility of other structural heart diseases cannot be ruled out.

The use of evidence-based therapies improves clinical outcomes in patients with HF and reduced ejection fraction (HFrEF). As seen in our patient’s case, this decision was made to control the tachysystolic AF—several PVI procedures were performed. However, these procedures were ineffective with the recurrence of AF after each procedure. There are several reasons for AF recurrence after the PVI procedure, usually due to technical limitations, such as non-transmural lesions and anatomical gaps in ablation lines or in cases where AF is driven by non-pulmonary vein foci—including the superior vena cava (SVC), coronary sinus, left atrial posterior wall, etc. [13] Moreover, our patient’s medical treatment prior to the cardiologist consultation was not optimized. Therefore, based on the 2021 ESC Guidelines for the diagnosis and treatment of acute and chronic heart failure, treatment with Sodium–Glucose Cotransporter-2 (SGLT-2) inhibitors was initiated to reduce the risk of CV death and hospitalizations in patients with HF with reduced EF [14]. Moreover, in 2023, the Focused Update on the above-mentioned guidelines was released, and recent studies have shown that SGLT-2 inhibitors can reduce the risk of HF hospitalizations and CV death in patients with both HFpEF or HFmrEF [15]. Moreover, treatment with an angiotensin receptor–neprilysin inhibitor (ARNI, sacubitril/valsartan) was initiated. In 2020, an observational study was performed, which studied patients with HFrEF and implantable devices treated with sacubitril/valsartan and reported a reduction in AF recurrence and burden [16]. In the PARADIGM-HF trial, sacubitril/valsartan was shown to be superior to enalapril in reducing hospitalizations for worsening HF, CV mortality, and all-cause mortality in patients with ambulatory HFrEF and LVEF ≤40% (which changed to ≤35% during the study) [17]. Additionally, the use of sacubitril/valsartan and SGLT-2i may allow for a reduction in loop diuretic requirement. Moreover, a 2019 study showed that the initiation of treatment with ARNI after stabilizing acute HF is feasible in patients with HFrEF [18], as implemented in our patient’s case. Mineral corticoid receptor antagonists (MRAs) are recommended in patients with HFrEF in addition to ACE-I and beta-blockers to reduce mortality and the risk of HF hospitalization and improve symptoms [15]. Moreover, in a systematic review and meta-analysis including 11 randomized clinical trials and 13 observational cohorts with 7914 patients in total, MRA-treated patients had significantly lower AF occurrence, with the greatest effect on recurrent episodes [19]. Subsequent to the improvements in HF treatment after three months, the patient’s echocardiographic data revealed an LVEF of 24%; however, it remained below 35%.

Moreover, the patient was uptitrated with beta-blockers; however, maximal admissible dosages were not achieved due to hypotension. Amiodarone was taken in the anamnesis, and it was not effective enough on rhythm control. However, digoxin was not prescribed for this patient as there is some data showing that digoxin does not reduce mortality rate in cases of HFrEF in combination with AF [20]. Therefore, without expecting the full medical treatment effect for controlling ventricular rate and HF symptoms, AV node destruction following a pacemaker upgrade to CRT were considered. The 2021 ESC Guidelines on cardiac pacing and cardiac resynchronization therapy indicate that patients with HFrEF who have received a conventional pacemaker or an ICD and subsequently develop worsening HF with a high proportion of RV pacing should be considered for ‘upgrade’ to CRT despite optimal medical treatment [21]. A meta-analysis performed in 2022, which was conducted on patient data from the Comparison of Medical Therapy, Pacing, and Defibrillation in Heart Failure (COMPANION) and Cardiac Resynchronization-Heart Failure (CARE-HF) trials, showed that CRT-P implantation for selected patients with HF can reduce mortality with guideline-approved HF pharmacological treatment. Moreover, the study shows that the most favorable sample includes patients with QRS duration > 140 ms and morphology of LBBB. However, observations do not exclude similar benefits if QRS is at least 120 ms. Our patient’s ECG revealed LBBB with a QRS duration of 122 ms [22]. Nevertheless, as the guidelines and performed studies show, individual cases with borderline QRS duration may be considered if the patient has worsening of HF symptoms and high RV pacing burden. In our case, the patient had deterioration of his HF symptoms, and optimizing his medical treatment was not enough to control tachycardia, so AV node ablation was considered, after which high RV pacing would be observed. However, there is a lack of large, randomized studies to show the advantages of AV node ablation following CRT implantation. In 2021, an APF-CRT multicenter randomized trial, which included 133 patients, revealed that AV junction ablation and CRT implantation in patients with permanent AF, narrow QRS, and at least one hospitalization reduced the risk of death from any cause during a follow-up of 4 years. It was hypothesized that the benefit was due to the combination of the rate control achieved by AV junction ablation with biventricular pacing [23]. Experts are in favor of the usefulness of CRT in patients with permanent AF and NYHA class III and IV [15]. In our patient’s case, an upgrade of the pacemaker to CRT following the destruction of the AV node was performed to control tachysystolic AF and worsening of HF. After the treatment adjustments, the patient’s symptoms regressed, and echocardiography showed an improved LVEF up to 41%.

## 4. Conclusions

AF can exacerbate HF and cause dysfunction. Novel treatment aligned with the latest guidelines can improve patient care and contribute to better symptom control. Medical treatment and implantable devices can help to control symptoms and reduce the risk of CV death or hospitalization due to HF risk.

## Data Availability

The original data presented in this study are available on reasonable request from the corresponding author. The data are not publicly available due to privacy concerns.
